# Rapamycin-filgrastim combination therapy ameliorates portal hypertension-induced splenomegaly: Role of β actin and S100A9 proteins modulation

**DOI:** 10.22038/IJBMS.2022.64034.14101

**Published:** 2022-06

**Authors:** Shaimaa A. Abdelrahman, Mohammed M. Abdelfatah, Akaber T. Keshta

**Affiliations:** 1 Medical Histology and Cell Biology Department, Faculty of Medicine, Zagazig University, Zagazig, Egypt; 2 Biochemistry Division, Chemistry Department, Faculty of Science, Zagazig University, Zagazig, Egypt

**Keywords:** Actins, Filgrastim, Hypertension, Portal, Sirolimus, Splenomegaly

## Abstract

**Objective(s):**

Thioacetamide (TAA) was administered to induce an animal model of liver disease with secondary splenomegaly to assess the mechanisms underlying the effects of rapamycin and filgrastim when taken separately or in combination on the biochemical and histopathological aspects of the liver and spleen.

**Materials and Methods:**

Thirty adult male albino rats were divided into five groups (control, TAA-treated group, TAA+rapamycin, TAA+filgrastim, and TAA+rapamycin+filgrastim group). We measured relative liver and spleen weights, serum levels of alanine transaminase (ALT), aspartate transaminase (AST), and albumin. Molecular docking modeling and histopathological examination of liver and spleen sections with hematoxylin and eosin and Masson trichrome staining with immunohistochemical detection of splenic CD3 and CD20 lymphocytes, S100A9 and β actin antibodies were detected. Morphometric and statistical analyses of the results were performed.

**Results:**

TAA administration altered the histological structure of the liver and spleen and impaired liver function. It increased the expression of splenic CD3, CD20 lymphocytes, and S100A9 while diminishing the expression of β actin. Each of rapamycin and filgrastim, when administered separately, improved liver and spleen indices and liver function, but rapamycin did not affect the albumin level. They lowered splenic B and T lymphocyte levels. Expression levels of S100A9 showed down-regulation while β actin levels were up-regulated when compared with TAA. Combination therapy improved liver and spleen tissue pathology and significantly ameliorated the expression of splenic lymphocytes through regulation of S100A9 and β actin expression.

**Conclusion:**

The synergistic effect of combination therapy was dependent on the regulation of splenic S100A9 and β actin levels.

## Introduction

Chronic liver diseases as a consequence of hepatitis virus infections with resultant liver fibrosis, cirrhosis, or hepatocellular carcinoma are prevalent in 80–85% of patients in Egypt (1). Obstruction of the blood flow returning from the spleen to the liver via its portal vein results in splenomegaly (2) where, the spleen acts as a large pool of blood, putting blood cells in prolonged contact with splenic macrophages and enzymes. This may result in cytopenia or pancytopenia which leaves patients with few or even no chances for many treatments that could be compatible with these conditions (3). Splenectomy was the treatment of choice to resolve this problem but it prevents the body from making use of one of the most important lymphatic organs (4). It also leaves patients with increased susceptibility to sepsis (5). 

Thioacetamide (TAA) is an organosulfur compound that was previously used as a fungicide but nowadays it is used to induce chronic liver diseases (fibrosis and cirrhosis) in experimental animal models (6).TAA was preferred to induce actual liver damage like fibrosis and cirrhosis more than the traditionally used CCl_4_, as it causes more liver damage and portal hypertension as a consequence (7). Therefore, in the present study, we used TAA to induce the animal model of chronic liver disease with subsequent splenomegaly.

Searching for new treatments, Mejias and Garcia-Pras (2) used rapamycin which showed good results in reducing spleen size but unfortunately did not improved platelet count. Rapamycin is a member of macrolide antibiotics, absorbed rapidly from the GIT due to its hydrophobic character. It reaches its sites of action mainly carried on its binding protein and then metabolized in the liver (8). Also, it is approved by the FDA as an immunosuppressive drug; therefore, it should be a safe and stable drug in this study (9). It is a multifunctional drug acting as an immunosuppressive (10), anti-rheumatoid arthritis and some types of cancers (11). Rapamycin binds to the FK506-binding protein (FKBP12) inside the cell forming a complex which, in turn, binds to the mTOR (mammalian target of rapamycin) complex and so performs its action. There are two known types of mTOR complexes which are mTORC1 and mTORC2. Rapamycin specifically binds to mTORC1. According to Li and Kim (12)**,** rapamycin’s effect on mTORC1 is modest and not very strong due to incomplete inhibition of the mTORC1-dependent pathways. To overcome these problems, it is advised to use another therapy besides rapamycin to block these subways and overcome single treatment limitations.

Filgrastim is a newly synthesized recombinant human methionyl granulocyte colony-stimulating factor (G-CSF), which stimulates proliferation and maturation of neutrophil progenitors and functional end-cell activation. It also facilitates their release into the blood (13). Filgrastim is mainly used for patients receiving chemotherapy due to common occurrence of neutropenia and cytopenia in these patients, thus they become more vulnerable to bacterial infections and associated complications (14). Patients receiving filgrastim could experience mild side effects such as abdominal pain, musculoskeletal pain, diarrhea, and headache. Severe complications such as splenic rupture, anaphylactic reaction, pulmonary hemorrhage, and hematuria/proteinuria were reported as rare side effects in patients using filgrastim for long periods (15).

To investigate the mechanisms underlying cellular growth, migration, and proliferation in the spleen, we studied the expressions of cell surface markers of B and T lymphocytes and the expression of β-actin and S100A9 proteins in detail in the different studied groups. β-actin is the most predominant actin isoform in non-muscular cells. It is reported to have a key role in cell motility and survival (16, 17). It is an important factor in fetus development because of its role in cell migration and growth. In the same context, absence of β-actin from T-cells by genetic modifications has altered their migration, ensuring the very important role of β-actin in cell motility (18).

Another protein called S100A9 which is a member of the S100 protein family was investigated in the present work. S100 proteins represent a category of multifunctional regulatory proteins involved in multiple forms of cellular actions. In order to complete their action, they usually bind to divalent cations such as calcium (Ca^2+^), copper (Cu^2+^), and zinc (Zn^2+^), but mainly bind to calcium so they are subgroups of Ca^2+^-binding EF-hand superfamily (19, 20). S100A9 was up-regulated in spleen protein extracts of animals with tumor or autoimmune encephalomyelitis, while spleens of normal mice showed low amounts of S100A9 (21). It is mainly found in granulocytes and monocytes besides its presence in activated keratinocytes (22). 

In the present study, we aimed to induce an animal model of splenomegaly secondary to portal hypertension induced by chronic TAA administration. We investigated the histopathological and biochemical changes in the liver and spleen after exposure to TAA and the role of administration of each of rapamycin or filgrastim either alone or in combination. Also, we aimed to clarify the possible mechanisms underlying the effects of combination therapy.

## Materials and Methods

***Chemicals***

*Thioacetamide (TAA)*

TAA (CAS No. 62-55-5, LOT No. L206471610, 98.5% extra pure) was purchased from Loba Chemie for laboratory reagents & fine chemicals. It was dissolved in sterile normal saline (0.9%) to be injected by the intraperitoneal route.

*Rapamycin*

Sirolimus≥99% purity was purchased from Alfa Aesar, Thermo Fisher Scientific Chemicals, Inc., Germany, product of USA. CAS No. 53123-88-9, stock number J62473, LOT: Y13D043. Imported by international Co. for scientific and medical supplies, Cairo, Egypt. Rapamycin was prepared by dissolving in (DMSO) nearly 250 mg/ml DMSO (23) to make a stock solution of rapamycin. The stock solution was mixed with a vehicle of 10% polyethylene glycol 6000 (PEG 6000) (24) and 2.5% tween 80 (25).

*Filgrastim*

Filgrastim Sedico® 300 µg/ml/vial, SEDICO pharmaceutical Co., Egypt, was purchased from a local pharmacy (CAS No. AJ05001A10100, LOT No. 0118113).

*Dimethyl sulphoxide (DMSO)*

99% purity, Batch No. L16A/0116/2511/13, was purchased from El-Gomhoureya Co. for trading medicines, chemicals & Medical appliances, Zagazig. Order No. 1001/120/3635. Manufactured by SDFCL - S D Fine-Chem Limited, India.

*Tween 80*

100% purity, Batch No. 2016/3, was purchased from El-Gomhoureya Co. for trading medicines, chemicals & Medical appliances, Zagazig. Manufactured by El Naser Pharmaceutical Chemical Co. (ADWIC).

*Polyethylene glycol 6000 (PEG-6000)*

Solid, CAS No. 25322-68-3, Batch No. 1212240916, was purchased from El-Gomhoureya Co. for trading medicines, chemicals & Medical appliances, Zagazig. Manufactured by research-lab fine chem. industries, India. Order No. 1001/120/3626/20025.

*Saline*

0.9% sterile saline for IV infusion was purchased from a local pharmacy.

***Animals***

Thirty adult male albino rats with a mean weight of 180 g were used in this study. Rats were obtained from the Faculty of science animal house at Zagazig University, Zagazig, Egypt. They were kept at 12 hr of light and dark cycles with free access to water and food. All animals were housed according to the guidelines for animal research issued by the National Institute of Health (26) and approved by the Animal Ethics Committee, Zagazig University (ZUIACUC) under number ZUIACUC/1/F/3/2018.

After two weeks of acclimatization, the animals were divided into five groups each with six rats as follows:

**Group I (control group):** Received a vehicle of tween 80 and PEG 6000 dissolved in saline by intraperitoneal injection at the same dose and duration as that of group III.

**Group II (TAA group):** Received TAA (dissolved in sterile normal saline 0.9%) by intraperitoneal injection at a dose of 200 mg/Kg/day, day after day (3 times a week) for 8 weeks (27).

**Group III (TAA+RAPA group):** Received TAA as group II followed by intraperitoneal injection of rapamycin at a dose of 2 mg/kg/day for 2 successive weeks (2).

**Group IV (TAA+FIL group):** Received TAA as group II followed by filgrastim administered by subcutaneous injection at a dose of 10 µg/kg every 48 hr for 2 successive weeks (28)

**Group V (TAA+RAPA+FIL group): **Received TAA as group II followed by intraperitoneal injection of rapamycin at a dose of 2 mg/kg/day, and at the same time along with rapamycin; filgrastim was administered by subcutaneous injection at a dose of 10 µg/kg every 48 hr. Both drugs were given for 2 successive weeks. 

***Methods***

By the end of the experiment, animals of all groups were anesthetized with ether inhalation. Blood samples were collected from the periorbital vein. Liver and spleen weights were detected for all groups after that, they were dissected for histopathological examination.

***Blood sampling***

The sera were separated and stored at -20 °C for alanine transaminase (ALT), aspartate transaminase (AST), and albumin determination according to Tietz (29).

***Molecular docking modeling***

Docking studies afford the most complete possible view of drug-target binding and expect the mode of action of active components. Physical binding with one or more cellular target proteins is well-known to result in biological activities of chemical compounds (30).

The 3D structures of rapamycin (RAPA) and filgrastim (FIL) into β-actin and S100-A9 proteins were recovered from RCSB Protein Data Bank (http://www.rcsb.org/pdb) (PDB ID code: 3QX3). The 2D structure of RAPA and FIL was captivated by ChemDraw. Then, the protonated 3D was constructed using standard bond angles and lengths, with the MOE 10.2015 software, subsequent geometry optimization and energy minimization were performed to utilize the Conf Search module in MOE, then the MOE file was collected to be accessible for the docking procedure. All minimizations were done with MOE until an RMSD incline of 0.05 kcal mol 1Å1 with the MMFF94x force field and the partial charges were automatically expressed (31).

The structures of the β-actin and S100-A9 were formulated for molecular docking via the Protonate 3D protocol in MOE by removing water molecules and the original ligand from the crystal structure of the protein, and by inserting H-atoms into the system with their standard geometry. Firstly, the confirmation procedures were validated by re-docking of the original lead ligand (etoposide), monitored by docking of RAPA and FIL into the active site after deleting the co-crystallized ligand (etoposide). Docking of the objective components was done using MOE-Dock. The MDB files of ligands to be deducted were packed and docking estimates were moved inevitably. The acquired presents were analyzed, and the models indicated the greatest ligand-enzyme bindings were chosen and collected for energy estimates.

***Tissue sampling***

Specimens of liver and spleen tissues were collected and prepared for light microscope examination at the Medical histology and cell biology Department, Faculty of Medicine, Zagazig University. The liver and spleen were fixed in Bouin’s solution, dehydrated, and embedded in paraffin wax. 5 μm thick sections were stained with hematoxylin and eosin (H&E) for routine histological examination, and Masson trichrome staining of liver sections for detection of collagen fiber deposition (32).

***Immunohistochemical study***

Immunohistochemical detection of CD3, CD20, S100A9, and β-actin antibodies was performed on spleen sections using the streptavidin-biotin complex immunoperoxidase technique. Sections were deparaffinized on charged slides and then incubated in 0.1% hydrogen peroxide for 30 min to block the endogenous peroxidase activity then, they were incubated overnight with the corresponding primary antibodies as follows:

***For CD3 immunoexpression***

Ready-to-use Polyclonal Rabbit Anti-Human CD3 (Code GA503, Dako Denmark A/S- Produktionsvej 42 -DK-2600 Glostrup – Denmark).

***For CD20 immunoexpression***

CD20 Polyclonal Rabbit IgG Antibody (Catalog # PA5 16701, dilution 1:300, Thermo Fisher Scientific, Rockford, USA).

***For S100A9 immunoexpression***

S100A9 Rabbit Polyclonal Antibody (Catalog # PA1-46489, Thermo Fisher Scientific, Rockford, USA).

***For β-Actin immunoexpression***

Mouse beta Actin Monoclonal Antibody (AC-15) (Product # MA1-91399, dilution 1:1,000, Thermo Fisher Scientific, Rockford, USA). 

 After several washings with PBS, sections were incubated with secondary antibody (biotinylated goat anti-rabbit or anti-mouse IgG, Zymed Laboratories, South San Francisco, CA, USA) for 30 min at room temperature then with streptavidin–peroxidase conjugate. After that, sections were washed with PBS and incubated with diaminobenzidine (DAB; Sigma–Aldrich, St. Louis, MO, USA) for 5 min and counterstained with Mayer’s hematoxylin. For negative controls, the primary antibodies were replaced by PBS (33)**.**

***Image analysis and morphometric study***

Measuring portal vein diameter in the studied groups in addition to the area percent (area %) of liver collagen fibers and the immunohistochemical reaction of CD3, CD20, S100A9, and β-actin antibodies were morphometrically analyzed using an image analyzer computer system. The data were obtained using a Leica Qwin 500 image analyzer computer system (Cambridge, UK, Leica Microsystems Imaging Solutions Ltd) in the image analyzing unit at the Pathology Department, Faculty of Dentistry, Cairo University. The image analyzer consisted of a color video camera, color monitor, and hard disc of IBM personal computer connected to the Olympus microscope (CX 41) and controlled by Leica Qwin 500 software. The image analyzer was first calibrated automatically to convert the measurement units (pixels) produced by the image analyzer program into actual micrometer units. These measurements were done using an objective lens of magnification 40, i.e., of total magnification of 400. Ten readings were obtained for each specimen.

***Statistical analysis***

The data obtained (relative liver and spleen weights, serum levels of ALT, AST, and albumin, and morphometric results) for all groups were expressed as means and standard deviations (SD) and subjected to statistical analysis using one-way analysis of variance (ANOVA) test for comparison between the different studied groups (more than two groups). The least significant difference (LSD) test was also done to find significance between groups (34). An IBM computer with SPSS version 20 was used for these calculations.

## Results

***Molecular Docking Study***

**Figure 1:**
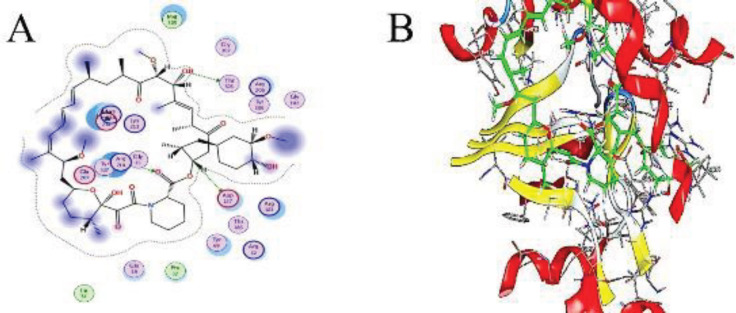
(A) 2D molecular docking of rapamycin and Beta-Actin. (B) 3D molecular docking of rapamycin and Beta-Actin (PDB: 2BTF, A chain)

To better understand the probable binding modes, a molecular docking study was carried out for both rapamycin (RAPA) and filgrastim (FIL) into β-actin and S100-A9 proteins. The study revealed that the co-crystalline of RAPA can fit in the active site pocket of β-actin with a docking score of -2.66 kcal.mol^-1^ and RMSD of 1.0933Å. It interacted with ASP 157, THR 303, and ARG 210 by H-bonding interaction in ranges of 2.78, 3.26, and 3.37 Å; respectively, with -1.0, -0.6, and -1.9 kcal.mol^-1^ as shown in (Table 1 and Figure 1). RAPA can interact with the active amino acid residues with H-bonding with good docking scores. Because of the lowest binding energies ranging, the interactions between rapamycin and Beta-Actin were quite favorable.

**Figure 2:**
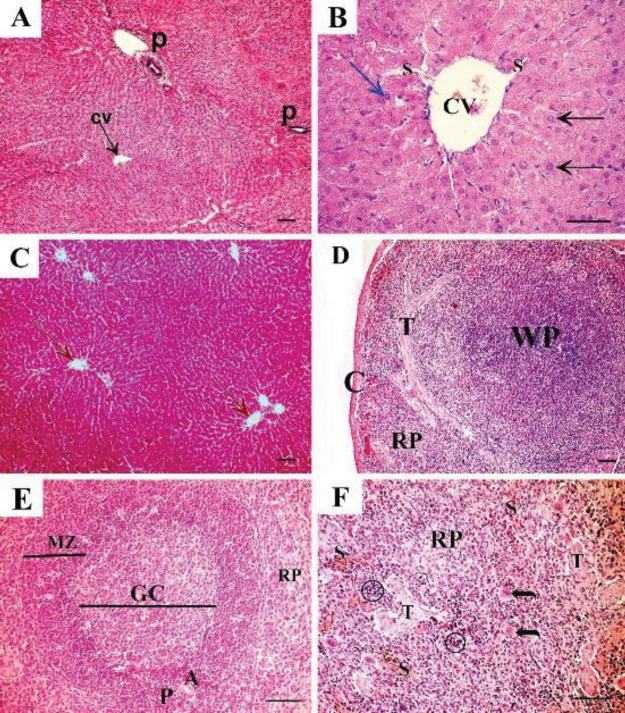
Control group shows A: H&E-stained liver section showing polygonal classic hepatic lobules with hepatocytes arranged in cords and radiating from central veins (CV). The portal area (P) is noticed at the angles of adjacent lobules. B: The higher magnification of the same group shows polygonal hepatocytes with rounded vesicular nuclei and acidophilic cytoplasm (black arrow). Some cells are binucleated (blue arrow). Blood sinusoids lined with endothelial cells (S) are seen between hepatocytic cords. Masson trichrome-stained liver sections show. C: Masson trichrome-stained liver section shows minimal amount of collagen deposition around the central vein and portal area in the control group. D: H & E-stained section of control rat spleen showing normal spleen architecture with its two major components: white pulp (WP) and red pulp (RP). Thin connective tissue capsule (C) and connective tissue trabeculae (T) can be seen. E: examined at higher magnification, the white pulp has lymphatic follicles with pale germinal center (GC) and a peripherally located central arteriole (A), surrounded by dark periarterial lymphatic sheath (P). Sharp marginal zone (MZ) separates the white pulp from the red pulp (RP). F: the red pulp (RP) of the same group shows fibrous connective tissue trabeculae (T) and splenic sinuses (S). Small basophilic darkly stained erythropoietic cells (circle) and megakaryocytes (curved arrow) can be seen. (A, C, D x10, scale bar 50 µm; B x40, scale bar 30 µm; E, F x20, scale bar 50 µm)

**Figure 3:**
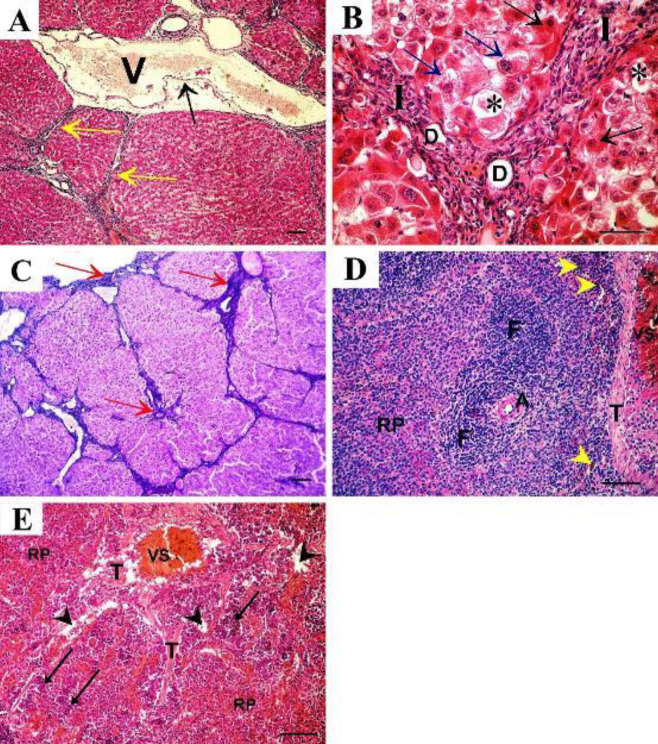
TAA-treated group shows A: H&E staining shows loss of the normal organization of hepatic lobules with fibrous tissue encapsulating the pseudo lobules (yellow arrows). Dilated and congested portal (P) vein lined with distorted endothelium showing separation and discontinuity (arrow) is also seen. B: Higher magnification shows pseudo lobules and fibrous tissue encapsulation with marked inflammatory cell infiltration (I). Pseudobile ducts (D) can be seen in the fibrous septa. Some hepatocytes show dark acidophilic cytoplasm with small dark nuclei (black arrow). Necrotic hepatocytes with vacuolated cytoplasm (blue arrow) and some cells that lost nuclei with marked cytoplasmic degeneration (asterisks) can be seen. C: Masson trichrome staining shows thickened connective tissue capsule with increased thickness of septa between lobules. D: spleen sections (H&E) show multiple lymphatic follicles (F) indicating hyperplasia of white pulp with thickened central arteriole (A). Hypercellular red pulp (RP) containing plenty of hemosiderin-laden macrophages (yellow arrowhead), thickened trabeculae (T), and congested venous sinus (VS) could be seen. E: Other sections of the same group show areas of separation (arrowhead) indicating edema of the red pulp. Aggregates of erythropoietic cells (arrow) are scattered throughout the hypercellular red pulp (RP). Disorganized connective tissue trabeculae (T) with congested venous sinus (VS) could be seen (A, C x10, scale bar 50 µm; B x40, scale bar 30 µm; D, E x20, scale bar 50 µm)

**Figure 4:**
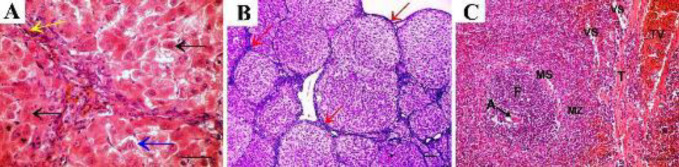
Rapamycin-treated group shows. A: fibrous tissue septa between hepatic lobules (yellow arrow), congested sinusoids (S), some hepatocytes show acidophilic cytoplasm with vesicular nuclei (black arrow) and other cells show cytoplasmic vacuolations (blue arrow). B: Thickened connective tissue capsule with increased thickness of septa between lobules in Masson trichrome staining. C: Lymphatic follicles with thickened abnormal shaped arteriole (A), marginal sinus separates the follicle from the hypertrophied marginal zone (MZ). Dilated venous sinuses (VS) in hypercellular red pulp. Disorganized connective tissue trabeculae (T) with congested trabecular vein (TV). (A x40, scale bar 30 µm; B x10, C x20, scale bar 50 µm)

Also, protein-protein interaction docking results showed interaction of FIL with β-actin, S100-A9 protein, CD3 receptor, and CD20 with docking scores of -244.89, -234.65, -327.69, and -331.57, kcal.mol^-1^, respectively, and RMSD of the interference residues within 5.0 Å from their interacting partner or each other, and the corresponding distances as shown in Tables 1-4S and Figures 1-4S.

***Liver and spleen sizes***

As shown in Table 2, TAA administration resulted in nearly a 124% increase in spleen size compared with negative control rats (expressed by spleen/body S/B ratio). A concomitant enlargement in liver size was evolved by nearly 69% more than the control group. Spleen size was decreased by 35.7% when the positive control group was treated with RAPA alone. FIL treatment reduced spleen size by 13%. Combined treatment markedly reduced splenomegaly by 40.8%. Liver size also decreased in splenomegaly groups nearly by the same percent, around 20%, with no preferred group.

***Liver function tests***

As shown in Table 3, Alb slightly decreased by 1.6% in TAA treated group, due to liver intoxication by TAA. Further decrease continued in case of treatment with RAPA alone by 20.6% compared with the positive control group, while it was less with FIL treatment (RAPA effect on Alb synthesis). Combination therapy, RAPA, and FIL showed a less potent decrease than RAPA alone,10.4% (FIL ameliorated RAPA effect).

Blood levels of ALT and AST were significantly increased by 42.1% and 44.2%, respectively in the TAA group. Treatment of splenomegaly rats by RAPA was found to reduce ALT and AST which gives the indication that the liver became better with more AST reduction by 28.8% than ALT 7.4%, due to the shorter half-life of AST compared with ALT. Treating splenomegaly rats with FIL decreased ALT by 20.9% and 30.2% for AST. The combination therapy protocols showed a highly significant decrease (*P*<0.001) in serum levels of liver enzymes compared with the TAA group.

***Histopathological examination of liver and spleen tissues***

Control adult male albino rats’ liver revealed polygonal classic hepatic lobules in close contact with each other. Each lobule was formed of cords of hepatocytes radiating from the central vein. The portal areas were noticed at the corners of adjacent hepatic lobules (Figure 2A). At a higher magnification, polygonal hepatocytes showed vesicular nuclei and acidophilic cytoplasm, and some cells were binucleated. Blood sinusoids lined with endothelial cells were seen between hepatocytic cords (Figure 2B). Examination of Masson trichrome-stained sections revealed a minimal amount of collagen deposition around the central vein and portal area (Figure 2C).

The spleen of the control group revealed normal spleen architecture with its two major components; white pulp and red pulp covered by thin connective tissue capsule (Figure 2D). The white pulp was composed of lymphatic follicles with a pale germinal center and a peripherally located sharp marginal zone. Central arteriole surrounded by dark periarterial lymphatic sheath was seen at the periphery of the follicle (Figure 2E). The red pulp showed connective tissue trabeculae and splenic sinuses and contained small basophilic darkly stained erythropoietic cells and megakaryocytes (Figure 2F).

***Effect of chronic TAA administration on the liver and splenic tissues***

Thioacetamide-treated (TAA) group showed loss of the normal organization of hepatic lobules with fibrous tissue encapsulation. Dilated and congested portal vein lined by distorted endothelium showed separation and discontinuity (Figure 3A). At a higher magnification, histopathological changes were obvious in the form of pseudo lobules formation with marked inflammatory cell infiltration. Pseudo bile ducts could be seen in the fibrous septa. Some hepatocytes showed dark acidophilic cytoplasm with small dark nuclei. Necrotic hepatocytes with vacuolated cytoplasm were evident. Other hepatocytes lost nuclei with marked cytoplasmic degeneration (Figure 3B). Thickened connective tissue capsules with increased thickness of septa between lobules were evident by Masson trichrome staining (Figure 3C)

The spleen of the same group showed hyperplasia of white pulp with thickened central arteriole. Hypercellular red pulp containing plenty of haemosiderin-laden macrophages, thickened trabecula, and congested venous sinus were evident (Figure 3D). The red pulp showed edema, hypercellularity, and scattered aggregates of erythropoietic cells in addition to disorganized connective tissue trabeculae (Figure 3E). 

***Effects of rapamycin or filgrastim monotherapy on TAA-exposed liver & spleen***

**Figure 5:**
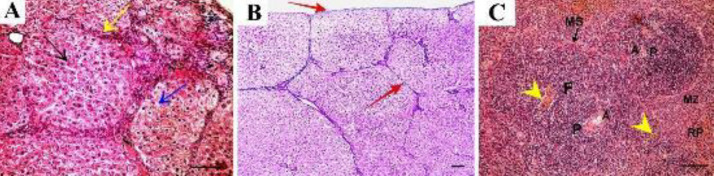
Filgrastim-treated group shows A: Fibrous tissue septa between hepatic lobules (yellow arrow), congested sinusoids (S), some hepatocytes show acidophilic cytoplasm with vesicular nuclei (black arrow) and other cells show cytoplasmic vacuolations (blue arrow). B: Thin connective tissue capsule and septa between liver lobules. C: A splenic section shows lymphatic follicle (F) with peripheral dilated arteriole (A) surrounded by periarterial lymphatic sheath (P), hemosiderin-laden macrophages (yellow arrowhead) could be seen in the follicle. The marginal sinus (MS) separates the follicle from the marginal zone (MZ). Normal shaped red pulp (RP) can be seen. (A x40, scale bar 30 µm; B x10, C x20, scale bar 50 µm)

The groups treated with either rapamycin or filgrastim alone showed fibrous tissue septa between hepatic lobules, congested sinusoids, and some hepatocytes appeared normal with acidophilic cytoplasm and vesicular nuclei, but other cells showed cytoplasmic vacuolations (Figures 4A&5A, respectively). Thickened connective tissue capsule and septa between lobules were evident in the rapamycin-treated group (Figure 4B). The filgrastim-treated group showed thin connective tissue capsules and septa between liver lobules (Figure 5B). 

On the spleen, the rapamycin-treated group showed lymphatic follicles with thickened abnormal shaped arteriole, hypertrophied marginal zone, dilated venous sinuses, hypercellular red pulp, and disorganized connective tissue trabeculae with congested trabecular vein (Figure 4C). The filgrastim-treated group showed lymphatic follicles with peripheral dilated arteriole, Haemosiderin-laden macrophages could be seen in the follicle. Normal shaped red pulp could be seen (Figure 5C).

***Effect of combination therapy on TAA-exposed liver & spleen***

**Figure 6:**
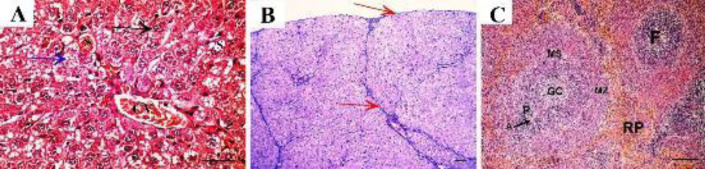
Combined therapy shows A: Normal appearance of liver lobule and hepatocytes (black arrow), but some of them still show vacuolations (blue arrow). The central vein (CV) and sinusoids (S) are congested. B: Minimal amounts of collagen fibers are observed in the capsule and septa of the combined treatment group (arrows indicate collagen deposition). C: The spleen of the same group shows lymphatic follicles (F) with a pale germinal center (GC) and a peripherally located central arteriole (A), surrounded by a dark periarterial lymphatic sheath (P). The marginal sinus can be seen separating the lymphatic follicle from the sharp marginal zone (MZ). The red pulp (RP) shows normal appearance (A x40, scale bar 30 µm; B x10, C x20, scale bar 50 µm)

Combined therapy showed marked amelioration in the appearance of liver lobule and hepatocytes but some of them still had vacuolations. The central vein and sinusoids were congested (Figure 6A). Minimal amounts of collagen fibers were observed in the capsule and septa of the combined treatment group (Figure 6B). On the spleen, combination therapy showed normal shaped lymphatic follicles with pale germinal center and normal shaped central arteriole, surrounded by a dark periarterial lymphatic sheath. The marginal sinus separates the lymphatic follicle from the sharp marginal zone. The red pulp showed normal appearance (Figure 6C).

***Expression of splenic lymphocytes in different groups***

**Figure 7:**
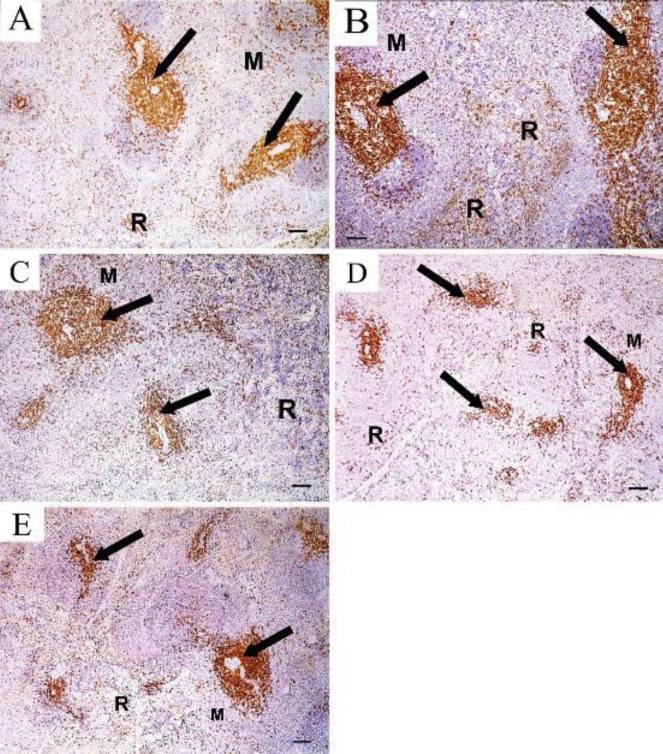
CD3 immunostained sections in the spleen showing A: Control group shows CD3+ T cells appearing mainly in the periarterial lymphatic sheath (arrow) of the white pulp, some cells can be seen in the marginal zone (M) and red pulp (R). B: TAA group shows a marked increase in the number of CD3+ T cells in the PALS (arrow), marginal zone (M), and red pulp (R). C: Rapamycin-treated group showing an apparent increase in the number of CD3+ T cells in PALS (arrow), marginal zone (M), and red pulp (R). D: Filgrastim-treated group showing normal appearance of CD3+ T cells in the PALS (arrow), marginal zone (M), and red pulp (R). E: combined rapamycin and filgrastim treatment group showing normal appearance of CD3+ T cells in the PALS (arrow), marginal zone (M), and red pulp (R). (Immunoperoxidase technique for CD3 x 10, scale bar 50 µm)

CD3+ T cells of the control group appeared mainly in the periarterial lymphatic sheath (PALS) of the white pulp. Few cells were seen in the marginal zone and red pulp (Figure 7A). TAA group showed a marked increase in the number of CD3+ T cells in the PALS of the white pulp, also in the marginal zone and red pulp in comparison to the control group (Figure 7B). The rapamycin-treated group showed an apparent increase in the number of CD3+ T cells in PALS, marginal zone, and red pulp compared with the control group (Figure 7C). The filgrastim-treated group showed normal appearance of CD3+ T cells in PALS, marginal zone, and red pulp (Figure 7D). The combined rapamycin and filgrastim treatment group showed normal appearance of CD3+ T cells in PALS, marginal zone, and red pulp (Figure 7E).

**Figure 8:**
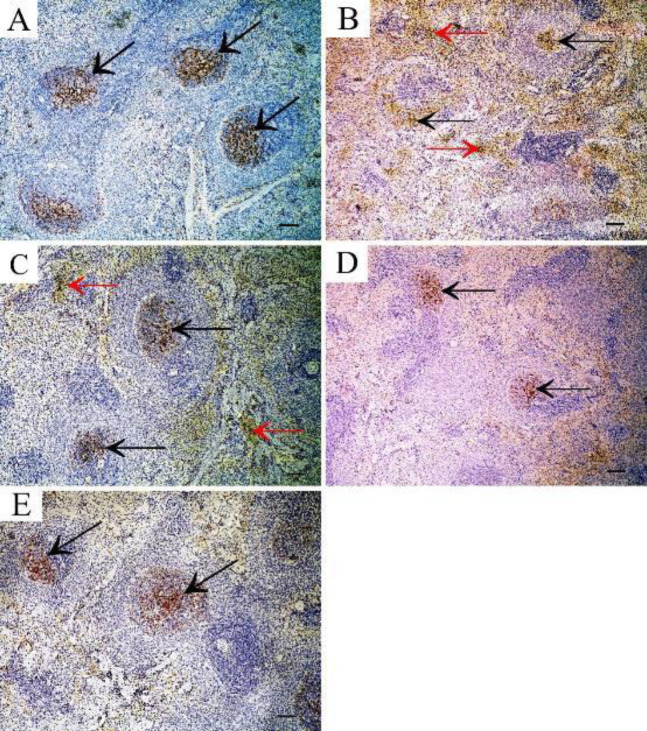
CD20 immunostained sections in the spleen showing A: control group shows CD20+ B cells appearing mainly in lymphatic follicles of white pulp (arrow). B: TAA-treated group showing increased CD20+ B cells in lymphatic follicles of white pulp (black arrow) and in red pulp (red arrow). C: rapamycin-treated group showing CD20+ B cells in lymphatic follicles of white pulp (black arrow) and few in the red pulp (red arrow). D: Filgrastim-treated group showing CD20+ B cells in lymphatic follicles of white pulp (arrow). E: combined filgrastim and rapamycin-treated group showing CD20+ B cells in lymphatic follicles of white pulp (arrow) (Immunoperoxidase technique for CD20 x 10, scale bar 50 µm)

CD20+ B cells of the control group appeared mainly in lymphatic follicles of white pulp (Figure 8A) while, the TAA-treated group showed markedly increased expression of CD20+ B cells both in lymphatic follicles of white pulp and in red pulp (Figure 8B). The rapamycin-treated group showed expression of CD20+ B cells in lymphatic follicles of white pulp and few cells appeared in red pulp (Figure 8C). On the other hand, nearly normal appearance of CD20+ B cells localized in lymphatic follicles of white pulp was seen in spleen sections of the filgrastim-treated group (Figure 8D) and combined filgrastim and rapamycin group (Figure 8E).

***B-actin and S100A9 expression in different groups***

**Figure 9:**
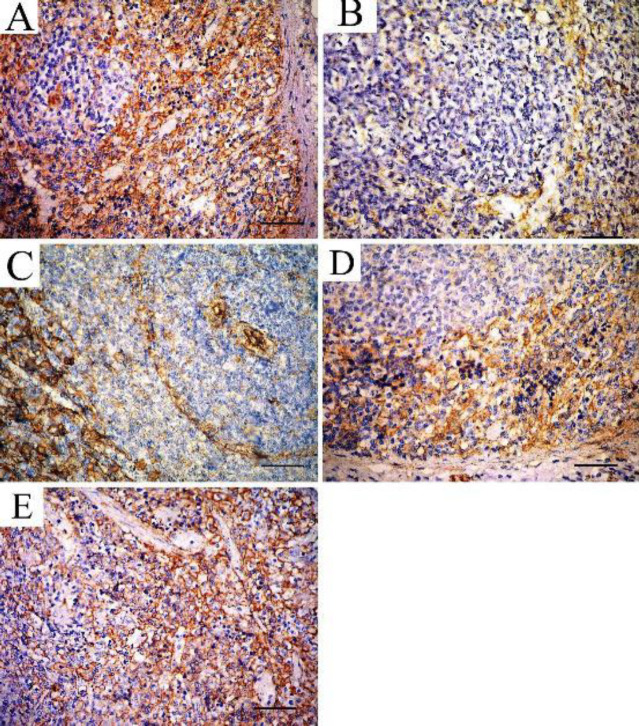
B-actin immunostained sections in the spleen showing A: Control group shows strong positive reaction in red and white pulps. B: TTA-treated group showing weak reaction in red and white pulp. C: Rapamycin-treated group showing weak reaction in the red and white pulp. D: Filgrastim-treated group showing strong positive reaction. E: Combined rapamycin and filgrastim group showing strong positive reaction (Immunoperoxidase technique for B-actin x40, scale bar 30 µm)

Immunohistochemical staining of B-actin antibody in the spleen sections of the control group showed strong positive reaction in red and white pulp (Figure 9A) while a weak reaction was observed in sections of the TTA-treated group (Figure 9B) and rapamycin-treated group (Figure 9C), when compared with control group. The filgrastim-treated group (Figure 9D) and combined rapamycin and filgrastim group (Figure 9E) showed a strong positive reaction. 

**Figure 10:**
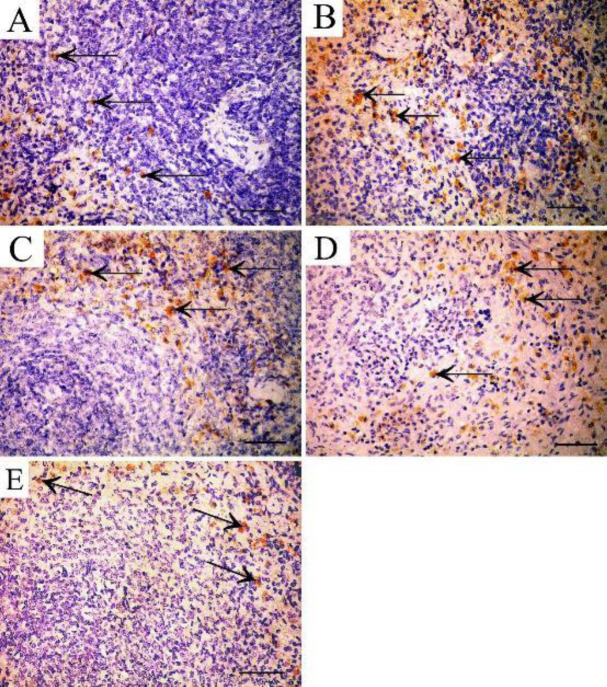
S100A9 immunostained section in the spleen showing A: Control group shows positive cytoplasmic immunoreaction for S100 antibody in some cells of the red pulp (arrow). B: TAA group showing increased cytoplasmic immunoreaction in cells of red and white pulp (arrow). C: Rapamycin-treated group showing increased cytoplasmic immunoreaction in cells of red pulp (arrow). D: Filgrastim-treated group showing positive cytoplasmic immunoreaction in some cells of the red pulp (arrow). E: Combined rapamycin and filgrastim-treated group showing positive reaction in few cells of red pulp (arrow) (Immunoperoxidase technique for S100 x 40, scale bar 30 µm)

Immunohistochemical-stained sections of S100 antibody in the spleen of the control group showed positive cytoplasmic immunoreaction in some cells of red pulp (Figure 10A), while the TAA-treated group showed increased cytoplasmic immunoreaction in cells of red and white pulp when compared with control group (Figure 10B). The rapamycin-treated group also showed increased cytoplasmic immunoreaction for S100 antibody in cells of red pulp (Figure 10C). The filgrastim-treated group showed positive cytoplasmic immunoreaction in some cells of red pulp (Figure 10D). Combined rapamycin and filgrastim-treated group showed a positive reaction in a few cells of red pulp (Figure 10E).

***Morphometric and statistical results (***Table 4***)***

TAA treatment showed a highly significant increase (*P*<0.00001) in portal vein diameter (2.95±0.25) compared

with control (1.35±0.19). Both rapamycin (2.88±0.43) and filgrastim (2.72 ±0.44) treatment alone reduced portal vein diameter but it showed non-significant change with TAA and was significant with control. Combination therapy showed a highly significant (*P*<0.00001) decrease (1.92±0.38) in portal vein diameter compared with TAA, rapamycin, or filgrastim groups and was non-significant with the control group.

The TAA-treated group showed a highly significant increase (*P*<0.00001) in the mean area % of liver collagen fibers compared with the control. Treatment with either rapamycin or filgrastim alone showed a highly significant decrease in the mean area % of liver collagen fibers compared with TAA, but it was still significant with control. Combination therapy markedly reduced collagen fibers that were highly significant with TAA, rapamycin, and filgrastim and non-significant with control. 

The TAA-treated group showed a highly significant increase (*P*<0.001) in the mean area % of CD3, CD20, and S100A9 expression, and a highly significant decrease (*P*<0.001) in the expression of B-Actin compared with control.

Rapamycin treatment alone showed a highly significant difference in levels of CD3, CD20, and B-Actin expression compared with both control and TAA groups, while a highly significant difference was observed with the TAA group concerning S100A9 levels (more improvement). 

Filgrastim treatment alone showed a highly significant difference in levels of CD3, CD20, and S100A9 expression compared with TAA (indicating more improvement), regarding B-Actin, highly significant differences with control, TAA and RAPA were detected.

Combined treatment showed a highly significant difference with the TAA group concerning CD3 and CD20. A highly significant difference was also observed with TAA-group, rapamycin group, and filgrastim group while insignificant with control regarding S100A9 levels. Concerning B-Actin expression, it showed a highly significant difference with the TAA group and the filgrastim group.

## Discussion

Splenomegaly is one of the most common complications of liver disease especially portal hypertension and infectious HCV (35). In the present study, we induced an animal model of splenomegaly via chronic thioacetamide (TAA)-administration to mimic hepatic patients suffering from splenomegaly. Chronic TAA injection significantly increased liver weight by about 69% more than control. It can be explained by increased fibrogenesis and excess collagen fibers deposition in the interlobular spaces together with hepatocytes hypertrophy and portal vein dilation and congestion (36). In addition, extensive tissue damage with TAA with pseudo lobules formation and fibrous tissue encapsulation, also marked inflammatory cell infiltration were noticed. Hepatocytes showed evidence of both apoptosis (dark acidophilic cytoplasm with small dark nuclei) and necrosis (degenerated and vacuolated cytoplasm with lost nuclei). TAA’s effect on liver cells is likely due to its oxidative stress via generating reactive oxygen species (ROS) (37, 38). 

Biochemical analysis also ensured hepatic function impairment due to significant increase in serum levels of both ALT and AST (36.3% and 41.1%, respectively) and decreased serum albumin level by 1.6% compared with the control group. These findings emphasize TAA hepatotoxicity according to Lin *et al*. (39). Mir *et al*. (40) attributed the decreased levels of total proteins and albumin in the hepatotoxic group to disturbances in the carbohydrates, lipid, and protein metabolism in addition to decreased ribosomal RNA in TAA-induced cirrhotic liver. Hadeer and AL-Kaisie (37) also found increased serum levels of ALP, ALT, AST, and bilirubin in male mice after 2, 4, and 6 months of TAA toxicity. It caused centrilobular necrosis, fibrosis, microabscesses, and liver cirrhosis**.**

Splenomegaly with TAA was proven by a significant increase in the spleen size (by 124% more than control) with 42% more increase than that described in the study by Mejias and Garcia-Pras (2). Histopathological examination revealed hyperplasia of the white pulp, hypercellularity, and edema of the red pulp, thickened trabeculae, and congested venous sinuses. Our findings agreed with that of Chen *et al*. (41). 

Rapamycin-treated rats showed reduced liver weight (liver/body index decreased by 23.6% compared with the TAA group). Serum levels of ALT and AST were also decreased by 10.5% and 22.2%, respectively, which could be explained by hepatocyte regeneration, in addition to mTOR1 inhibition that reduced cellular apoptosis, so fewer enzymes were liberated from hepatocytes (42). Reduction of fibrosis was also noticed in liver sections of the same group due to inhibition of new collagen synthesis that follows mTOR inhibition and its collagenase activity on old collagens (43). Consequently, splenomegaly was markedly ameliorated (35.7% decrease compared with the TAA group) due to limited lymphocyte proliferation, angiogenesis, fibrogenesis, and inflammation in rats with PHT according to Chen *et al*. (41). Such amelioration was also attributed to the immunosuppressive and anti-proliferative properties of rapamycin in mammalian cells (12)**. **Wang *et al.* (9) added that early treatment with rapamycin effectively ameliorated the intrahepatic inflammation and fibrosis, improved liver function, and decreased splenomegaly and portal pressure. So, it seems that rapamycin treats the primary and the secondary problems evolved: liver cirrhosis and splenomegaly (2, 41).

Pharmacological inhibition of mTOR by rapamycin would have a wide range of clinical effects, so it can benefit patients with tumors while the use of rapamycin monotherapy in a broad spectrum of metabolic diseases is limited due to its modest efficacy. It could be attributed to the inability of rapamycin to completely block mTORC1-mediated signaling events. The presence of several feedback loops and up-regulation of compensatory pathways that promote cell survival and growth, represent obstacles facing rapamycin monotherapy. Thus, there is a critical need to develop new strategies that can overcome these drawbacks (12).

In the present study, when rapamycin was administered in combination with filgrastim, better improvement in liver and spleen weights occurred. In addition, improved liver function and lowered fibrogenesis were evident. Our findings were in accordance with Chou *et al*. (44), who attributed this amelioration to the synergistic effect of both drugs, also due to inhibition of the mTOR mechanism. Previous studies reported that liver regeneration is one beneficial outcome of filgrastim therapy, therefore it causes liver size to increase in normal rats and decrease in diseased rats (45). Filgrastim administration to normal rats was associated with increment in both liver and spleen sizes which was attributed to increased extramedullary hematopoiesis resulting in increased WBCs count (14, 46, 47). From the previous findings, we can say that filgrastim has opposite effects on splenic tissue depending on whether it is normal or enlarged. It behaves differently depending on whether the body is under stress, illness, or healthy.

In the present work, splenomegaly with TAA was associated with increased expression of splenic B and T lymphocytes as detected by immunohistochemical examination of CD20+ B- lymphocytes and CD3+ T-lymphocytes. Zheng *et al*. (48) attributed it to mTOR activation, and inhibition of this activation explains the down-regulation of lymphocyte levels with rapamycin treatment either alone or in combination with filgrastim according to Ye *et al*. (49). Moreover, rapamycin as an immunosuppressant acts by antiproliferation effect on lymphoid and non-lymphoid cells (50). It inhibits proliferation of both T- and B-cells via inhibition of IL-2 cytokine (10). Filgrastim also acts as an immunosuppressive agent like rapamycin on lymphocytes as mentioned by Martins* et al*. (51), therefore, more and more decrease in lymphocyte population in the splenic tissue when combined with rapamycin was shown in our results.

Studying in-depth the underlying mechanisms of action of both drugs, β-actin and S100A9 protein expression levels in splenic tissue were examined. They represent two kinds of different proteins found to have roles concerning cell motility inside different tissues, especially splenic tissue resident cells. They have opposing functions, while β-actin promotes cell migration, S100A9 inhibits this migration. Hence, any factor that increases the amount of β-actin or reduces S100A9 would result in promoting cell migration through a particular organ or tissue and vice versa (18, 21). Immunohistochemical findings in our study showed increased expression of S100A9 together with decreased β-actin expression with TAA administration. The dysregulation in the amount of these proteins explains abnormal lymphocyte trapping and hypercellularity in the spleen of the same group. Our findings were in accordance with Ishizuka *et al*. (52). Moreover, large amounts of S100A9 protein were previously detected in enlarged spleens of dogs with fibrohistiocytic nodules (53). Such findings provide a strong relationship between increased spleen size and S100A9 protein overexpression in spleen tissue. Moreover, researchers (54) proved that reduction in β-actin expression with TAA was due to direct reduction of its producing gene. 

Concerning rapamycin-treated rats, there was increased immunoexpression of β-actin which could be explained according to Love *et al*. (55), who stated that β-actin content did not respond to mTOR inhibition when they treated peripheral nerves under strain with rapamycin. They found decreased amount of mTOR after rapamycin treatment but with unexpected increase in β-actin content in the stressed nerves. This may be explained through the abolished inhibitory effect of mTORC1 on the PI3K after application of rapamycin (56). Another report about absence of rapamycin inhibitory effect when used alone on the amount of cellular actin was mentioned by *Speranza et al*. (57) on neuron cell elongation. There was other evidence that correlate rapamycin treatment with increased β-actin content in the HCC cell line, Human HCC cells Bel-7402, as described in Zhang *et al*. (58). Due to incomplete inhibition of mTORC1-dependent pathways by rapamycin, it may be compensated by other feedback loops which makes that blockade useless (12). Moreover, β-actin mRNA is one of non 50́-TOP mRNA (terminal oligopyrimidine mRNA) which makes β-actin less affected by mTORC1 activation or inhibition state (59). Contrary to these results, Eliseeva *et al*. (60) reported down-regulation of β-actin levels associated with inhibited mTOR signaling pathway with rapamycin. 

Our results showed that the expression of β-actin was improved with filgrastim treatment and a further increase was noticed in combined treatment, making cells’ locomotion ability favored and reflecting the extra reduction in spleen size. This could be attributed to the effect of G-CSF (filgrastim) on β-actin, being one of the anabolic cytokines. Our findings were in agreement with that of Izdebska *et al*. (61). They also supposed that G-CSF can bind to other similar receptors and induce increased F-actin content in other cell lines (G-CSF receptor-independent mechanism). This notice is very important because usually, lymphocytes lack these receptors, making these cells responsive to the G-CSF effect.

**Table 1:** Molecular docking results showing interaction between Rapamycin and Beta-Actin (PDB: 2BTF, A chain)

	**Ligand**	**Receptor**	**Interaction**	**Distance**	**E (kcal/mol)**	**Docking score**	**RMSD**
**Compound**	C20	OD1 ASP 157 (A)	H-donor	2.78	-1.0	-2.66	1.0933
	O 139	OG1 THR 303 (A)	H-donor	3.26	-0.6		
	O 71	NH1 ARG 210 (A)	H-acceptor	3.37	-1.9		

**Table 2:** Statistical results of relative liver and spleen weights among the studied groups

**Variable**	**Group**	**Mean**	**SD**	**Range**		**F**	**P**
**liver/body** **(g%)**	Control -ve	3.13	0.19	2.86	3.29	44.95	<0.001 **
	Control +ve (TAA)	5.29	0.44	4.80	5.86		
	TAA + rapamycin	4.04 a,b	0.37	3.66	4.59		
	TAA + filgrastim	4.65 a,b,c	0.27	4.32	5.00		
	TAA+rapamycin+filgrastim	4.24 a,b	0.21	4.03	4.55		
**spleen/body** **(g%)**	Control -ve	0.17	0.02	0.14	0.18	53.11	<0.001 **
	Control +ve (TAA)	0.37 a	0.04	0.34	0.45		
	TAA + rapamycin	0.24 a,b	0.02	0.22	0.27		
	TAA + filgrastim	0.32 a,c	0.05	0.27	0.41		
	TAA+rapamycin+filgrastim	0.22 a,b,d	0.01	0.20	0.23		

SD: Standard deviation, F: ANOVA test, **: Highly significant (*P*<0.001), *post hoc*: Tukey’s test, a: Significant with control –ve, b: Significant with control+ve (TAA), c: Significant with TAA + rapamycin, d: Significant with TAA + filgrastim

**Table 3:** Statistical results of liver function tests among the studied groups

Variable	Group	Mean	SD	Range		F	P
ALT (U/L)	Control -ve	63.00	5.87	54.00	70.00	98.20	<0.001 **
	Control +ve (TAA)	89.50 a	6.28	80.00	96.00		
	TAA + rapamycin	82.83 a	8.33	73.00	94.00		
	TAA + filgrastim	70.83 b,c	3.43	65.00	75.00		
	TAA+rapamycin+filgrastim	88.33 a,d	9.07	76.00	98.00		
AST (U/L)	Control -ve	157.50	14.73	145.00	182.00	28.71	<0.001 **
	Control +ve (TAA)	227.17 a	19.95	210.00	255.00		
	TAA + rapamycin	161.67 b	10.80	145.00	175.00		
	TAA + filgrastim	158.50 b	6.47	150.00	166.00		
	TAA+rapamycin+filgrastim	186.83 a,b,c,d	8.16	179.00	200.00		
Alb g/dl	Control -ve	3.40	0.09	3.30	3.50	56.82	<0.001 **
	Control +ve (TAA)	3.34	0.16	3.10	3.50		
	TAA + rapamycin	2.65 a,b	0.13	2.49	2.80		
	TAA + filgrastim	3.10 a,b,c	0.10	3.00	3.26		
	TAA+rapamycin+filgrastim	3.00 a,b,c	0.13	2.84	3.20		

Sd: Stander deviation, F: ANOVA test, **: Highly significant (*P*<0.001), *post hoc:* Tukey's test, a: Significant with control –ve, b: Significant with control+ve (TAA), c: Significant with TAA + rapamycin, d: Significant with TAA + filgrastim

TTA: thioacetamide

TTA: thioacetamide

TTA: thioacetamide

**Table 4:** Morphometry results (Mean ± SD of portal vein diameter and the mean area % of liver collagen fibers, CD3, CD20, β-ACTIN, and S100A9 immunoexpression among the studied groups)

**Variable**	**Group**	**Mean**		**SD**		**Range**					**F**		**P**
**Portal vein diameter (mm)**	Control -ve	1.35		0.19		1.1	1.6				23.97		< .00001**
	Control +ve (TAA)	2.95 a		0.25		2.6	3.2						
	TAA + rapamycin	2.88 a		0.43		2.3	3.4						
	TAA + filgrastim	2.72 a		0.44		2.2	3.3						
	TAA+rapamycin+filgrastim	1.92 b,c,d		0.38		1.4	2.4						
**Area % of liver collagen fibers**	Control -ve	1.43	0.26		1.1			1.8		62.94		< .00001**	
	Control +ve (TAA)	7.72 a	1.21		6.4			9.5					
	TAA + rapamycin	5.43 a, b	0.92		4.2			6.8					
	TAA + filgrastim	4.38 a,b	0.57		3.8			5.2					
	TAA+rapamycin+filgrastim	2.42 b,c,d	0.8		1.8			3.2					
**CD3** **(T-Cells)**	Control -ve	7.99		0.85		7.00			9.00		144.12		<0.001**
	Control +ve (TAA)	28.71 a		1.60		27.00			31.06				
	TAA + rapamycin	11.37 a,b		2.19		8.64			14.26				
	TAA + filgrastim	9.85 b		1.53		7.90			12.20				
	TAA+rapamycin+filgrastim	8.61 b		1.75		6.22			11.06				
**CD20** **(B-Cells)**	Control -ve	0.35		0.12		0.20			0.48		81.85		<0.001**
	Control +ve (TAA)	18.97 a		4.00		13.76			23.37				
	TAA + rapamycin	4.60 a,b		1.24		2.32			5.73				
	TAA + filgrastim	3.10 b		1.55		1.20			5.30				
	TAA+rapamycin+filgrastim	1.58 b		0.41		1.06			2.14				
**β-Actin**	Control -ve	40.36		4.08		35.36			45.78		110.32		<0.001 **
	Control +ve (TAA)	13.45 a		1.74		11.06			15.53				
	TAA + rapamycin	49.33 a,b		3.37		45.32			53.51				
	TAA + filgrastim	26.95 a,b,c		3.95		22.40			32.50				
	TAA+rapamycin+filgrastim	54.17 a,b,d		3.19		50.00			59.00				
**S100A9**	Control -ve	1.63		0.57		0.98			2.40		30.58		<0.001 **
	Control +ve (TAA)	4.56 a		0.89		3.79			5.77				
	TAA + rapamycin	2.35 b		0.48		1.70			2.84				
	TAA + filgrastim	2.12 b		0.50		1.50			2.90				
	TAA+rapamycin+filgrastim	0.90 b,c,d		0.18		0.60			1.07				

Sd: Standard deviation, F: ANOVA test, **: Highly significant (*P*<0.001), post hoc: Tukey's test, a: significant with control –ve, b: Significant with control+ve (TAA), c: Significant with TAA + rapamycin, d: Significant with TAA + filgrastim

TTA: thioacetamide

## Conclusion

Rapamycin monotherapy proved ameliorative on both liver and spleen in cases of splenomegaly secondary to portal hypertension, but less improvement was observed regarding serum albumin level. Filgrastim monotherapy showed better results regarding liver enzymes and albumin levels, increased splenic β- actin, and decreased S100A9 expression which have a prominent effect on lowering the levels of trapped lymphocytes in spleen tissue. The combination of the two drugs strongly improved the dysregulation in S100A9 and β actin levels, promoting cell migration of lymphocytes from spleen tissue and decreasing spleen size. Besides, combined treatment lowered liver fibrogenesis and improved liver function, so it also corrected the main cause of splenomegaly. By analyzing our results, we can conclude that the filgrastim-rapamycin combination could be promising in treating patients with splenomegaly secondary to liver disease with no need for splenectomy. More research is recommended to delineate other mechanisms underlying the action of both drugs in splenomegaly patients.

## Authors’ Contributions

SAA, MMA, and ATK contributed to study design and manuscript writing; MMA collected data and performed the practical part of the experiment and statistical analysis; SAA interpreted histological results and did image analysis; ATK interpreted the biochemical results; SAA and ATK analyzed and interpreted data, and supervised, directed, and managed the study and approved the final manuscript for publication.

## Funding

This research was self-funded by the authors and did not receive any specific grant from any funding agencies in the public, commercial, or not-for-profit sectors.

## Data Availability Statement

The data that support the findings of this study are available from the corresponding author upon reasonable request. 

## Conflicts of Interest

The authors declare that there are no conflicts of interest.
